# Cold sensing by Na_V_1.8-positive and Na_V_1.8-negative sensory neurons

**DOI:** 10.1073/pnas.1814545116

**Published:** 2019-02-12

**Authors:** A. P. Luiz, D. I. MacDonald, S. Santana-Varela, Q. Millet, S. Sikandar, J. N. Wood, E. C. Emery

**Affiliations:** ^a^Molecular Nociception Group, Wolfson Institute for Biomedical Research, University College London, London WC1E 6BT, United Kingdom

**Keywords:** cold, NaV1.8, Trpm8, GCaMP, nociception

## Abstract

The ability to detect environmental cold serves as an important survival tool. The sodium channels Na_V_1.8 and Na_V_1.9, as well as the TRP channel Trpm8, have been shown to contribute to cold sensation in mice. Surprisingly, transcriptional profiling shows that Na_V_1.8/Na_V_1.9 and Trpm8 are expressed in nonoverlapping neuronal populations. Here we have used in vivo GCaMP3 imaging to identify cold-sensing populations of sensory neurons in live mice. We find that ∼80% of neurons responsive to cold down to 1 °C do not express Na_V_1.8, and that the genetic deletion of Na_V_1.8 does not affect the relative number, distribution, or maximal response of cold-sensitive neurons. Furthermore, the deletion of Na_V_1.8 had no observable effect on transient cold-induced (≥5 °C) behaviors in mice, as measured by the cold-plantar, cold-plate (5 and 10 °C), or acetone tests. In contrast, nocifensive-like behavior to extreme cold-plate stimulation (−5 °C) was completely absent in mice lacking Na_V_1.8. Fluorescence-activated cell sorting (FACS) and subsequent microarray analysis of sensory neurons activated at 4 °C identified an enriched repertoire of ion channels, which include the Trp channel Trpm8 and potassium channel Kcnk9, that are potentially required for cold sensing above freezing temperatures in mouse DRG neurons. These data demonstrate the complexity of cold-sensing mechanisms in mouse sensory neurons, revealing a principal role for Na_V_1.8-negative neurons in sensing both innocuous and acute noxious cooling down to 1 °C, while Na_V_1.8-positive neurons are likely responsible for the transduction of prolonged extreme cold temperatures, where tissue damage causes pan-nociceptor activation.

## Significance

The cellular correlate for cold sensing has been ascribed to either Trpm8-expressing or Na_V_1.8-expressing neurons. Importantly, transcriptomic analysis shows that these neuronal populations are nonoverlapping. Using in vivo GCaMP imaging in live mice we show that the vast majority of acute cold-sensing neurons activated at ≥1 °C do not express Na_V_1.8, and that the loss of Na_V_1.8 does not affect acute cold-sensing behavior in mice. Instead, we show that cold-responding neurons are enriched with Trpm8 as well as numerous potassium channels, including Kcnk9. By contrast, Na_V_1.8-positive neurons signal prolonged extreme cold. These observations highlight the complexity of cold sensing in DRG neurons, and the role of Na_V_1.8-negative neurons in cold sensing down to 1 °C.

The ability to sense environmental cold serves as an essential survival tool. For more than a decade, numerous candidate proteins have been identified as putative cold sensors, mainly through loss-of-function studies where specific ion channels have been genetically deleted, or where specific populations of DRG neurons have been ablated. Of the candidates currently identified, the ion channel Trpm8, as well as the voltage-gated sodium channels Na_V_1.8 and Na_V_1.9, have all been associated with robust cold-insensitivity phenotypes. The genetic deletion of Trpm8 significantly attenuates cold sensitivity in mice, and ablation of the Trpm8-expressing population of neurons almost completely abolishes cold sensitivity down to 0 °C (1–4). In addition to these findings, Na_V_1.8, a sodium channel that is predominantly expressed in DRG neurons, has been shown to play a role in pain at low temperatures, owing to its relative insensitivity to cold-induced channel inactivation, and subsequent ability to propagate action potentials at temperatures as low as 10 °C (5). More recently, the loss of the sodium channel Na_V_1.9, which is mainly expressed within the Na_V_1.8-expressing population of DRG neurons, has been shown to significantly attenuate oxaliplatin-induced cold allodynia (6). Collectively, these studies suggest that both the Trpm8- and Na_V_1.8-expressing populations of DRG neurons are essential for noxious cold sensing. What is intriguing, however, is that recent single-cell RNA sequencing data from mouse DRG neurons show that the genes encoding Trpm8 and Na_V_1.8 show a little to no overlap (7). To further investigate this, we set out to define the distribution and identity of cold-sensitive DRG neurons, in live mice, using in vivo imaging.

## Results

### Distribution of DRG Sensory Neurons Responsive to Noxious Cold, in Vivo.

**Fig. 1.**
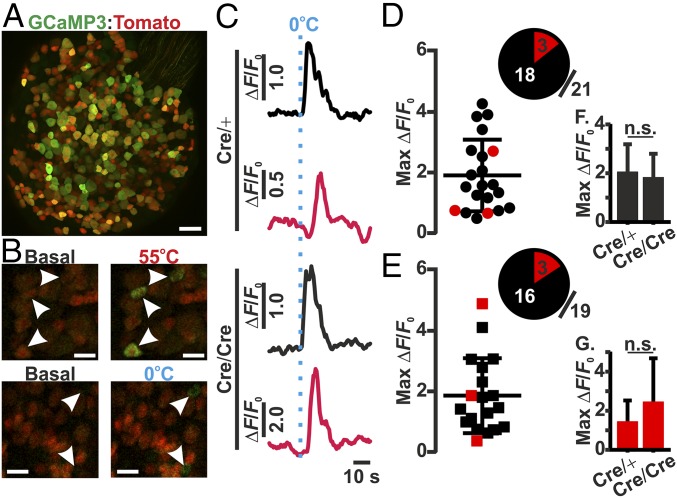
Cold-induced changes in GCaMP3 fluorescence from Na_V_1.8^Cre/+^ and Na_V_1.8^Cre/Cre^ null DRG neurons, in vivo. (*A*) High-resolution confocal image of an entire L4 DRG-expressing Pirt-GCaMP3 (pan-DRG GCaMP3; green), Na_V_1.8-Cre, and a Cre-dependent tomato reporter (red). (Scale bar, 100 μm.) (*B*) Representative images of basal and temperature-induced changes in GCaMP3 fluorescence recorded at the level of the DRG in response to ipsilateral plantar stimulation. (Scale bar, 50 μm.) (*C*) Changes in GCaMP3 fluorescence in response to 0 °C plantar stimulation, from cold-responsive Na_V_1.8^Cre/+^ (*Top*) and Na_V_1.8^Cre/Cre^ null (*Bottom*) DRG neurons. Traces are taken from nontomato (black) and tomato-expressing (red) neurons, respectively. (*D* and *E*) Summary of maximum fluorescent changes following 0 °C plantar stimulation of cold-responsive neurons from Na_V_1.8^Cre/+^ (*D*; *n* = 4) and Na_V_1.8^Cre/Cre^ null (*E*; *n* = 5) mice, showing the distribution and relative proportion of nontomato (black)- and tomato (red)-expressing neurons. (*F* and *G*) Average maximum changes in GCaMP3 fluorescence of either (*F*) nontomato- or (*G*) or tomato-expressing neurons in response to 0 °C plantar stimulation, from Na_V_1.8^Cre/+^ and Na_V_1.8^Cre/Cre^ null DRG neurons.

To identify cold-sensitive neurons in vivo, we used a previously developed in vivo imaging technique to study the responses of individual DRG neurons in situ (8). Mice coexpressing Pirt-GCaMP3 (which enables pan-DRG GCaMP3 expression), Na_V_1.8 Cre, and a Cre-dependent reporter (tdTomato) were used to investigate the relative distribution of cold-sensitive neurons within mouse DRG that innervate the sciatic nerve (lumbar regions L3–L5) (Fig. 1*A*). Importantly, Na_V_1.8 is haplosufficient, meaning that Na_V_1.8^Cre/+^ mice still retain normal Na_V_1.8 function and associated pain behaviors (9, 10). In contrast, Na_V_1.8^Cre/Cre^ null mice are equivalent to Na_V_1.8-null (Na_V_1.8^−/−^) mice, as Cre replaces both copies of the Scn10a gene, while keeping the promoter region intact; therefore, these mice retain no residual Na_V_1.8 function (9). The application of either 55 or 0 °C water to the ipsilateral hind paw caused the robust activation of discrete populations of thermosensitive sensory neurons within the mouse DRG (Fig. 1*B*). On average, between three and six cold-sensitive neurons were present within each imaging field. Interestingly, in Na_V_1.8^Cre/+^ mice, the majority of cold-sensitive neurons (18/21; 85.7%) did not express Na_V_1.8 as defined by reporter fluorescence (Fig. 1 *C* and *D*). When the same assessment was performed on Na_V_1.8^Cre/Cre^ null mice, the relative number and distribution of cold-sensitive neurons remained largely unchanged, with 84.2% (16/19) of neurons negative for Na_V_1.8 expression (Fig. 1*E*). Furthermore, there was no significant difference in the maximal cold response from sensory neurons, as measured by change in GCaMP3 fluorescence, between Na_V_1.8^Cre/+^ and Na_V_1.8^Cre/Cre^ null mice (Fig. 1 *F* and *G*). To investigate whether the expression of Cre was different between Na_V_1.8^Cre/+^ and Na_V_1.8^Cre/Cre^ null mice, the number of tdTomato-expressing DRG neurons from lumbar regions L3–L5 was quantified. The number of tdTomato-expressing neurons from either genotype did not differ significantly, with 81.28% (803/988) and 79.28% (941/1187) of neurons being observed in DRG from Na_V_1.8^Cre/+^ and Na_V_1.8^Cre/Cre^ null mice, respectively (*SI Appendix*, Fig. S1).

### Threshold-Specific Activation of Cold-Sensitive DRG Neurons, in Vivo.

**Fig. 2.**
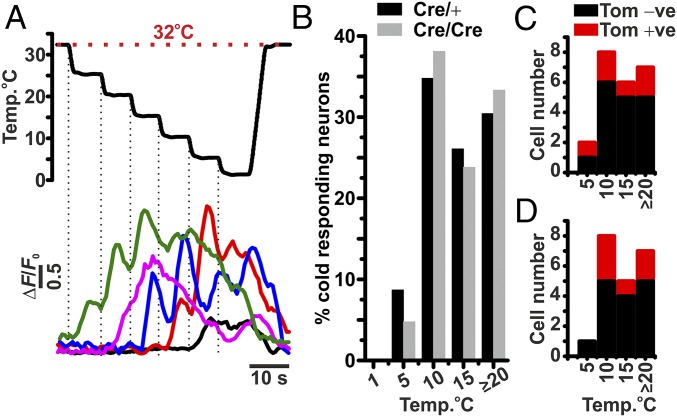
Threshold of activation of cold-sensitive neurons from Na_V_1.8^Cre/+^ and Na_V_1.8 ^Cre/Cre^ null DRG, in vivo. (*A*) Representative changes in GCaMP3 fluorescence from threshold-specific cold-responsive DRG neurons in response to a series of ∼−Δ5 °C changes in stimulus temperature, applied at the plantar surface. (*B*) Normalized summary of the threshold of activation of cold-sensitive DRG neurons from Na_V_1.8^Cre/+^ (*n* = 4) and Na_V_1.8^Cre/Cre^ null (*n* = 5) mice. (*C* and *D*) Summary of the number of cold-responsive neurons with or without tomato fluorescence, from Na_V_1.8^Cre/+^ (*C*) and Na_V_1.8^Cre/Cre^ null (*D*) DRG neurons.

Importantly, the stimulation of the plantar surface with 0 °C water is likely to activate all sensory neurons responsive to some degree of cooling. Therefore, we used a computer-controlled Peltier stimulation device (Medoc NeuroSensory Analyzer) to study the responses of threshold-specific cold-sensitive neurons. To investigate the activation thresholds of specific cold-sensitive neurons, we applied a cooling protocol that stepped down by ∼5 °C, at a rate of 8 °C.s^−1^, every 5 s, from an initial baseline of 32 °C. The application of this protocol identified several populations of cold-sensitive neurons, exhibiting discrete activation thresholds ranging from 25 to 5 °C (Fig. 2*A*). Interestingly, most of the cold-sensitive neurons had an activation threshold between 25 and 10 °C, with very few exhibiting a threshold <10 °C (Fig. 2*B*). As observed in the 0 °C water-stimulation experiments, the majority of cold-sensitive neurons identified were Na_V_1.8 negative, in both Na_V_1.8^Cre/+^ and Na_V_1.8^Cre/Cre^ null mice. Furthermore, the distribution of threshold-specific cold-sensitive neurons remained largely unchanged in Na_V_1.8^Cre/Cre^ null mice (Fig. 2 *C* and *D*).

### Coding Threshold-Specific Cold-Sensitive DRG Neurons, in Vivo.

**Fig. 3.**
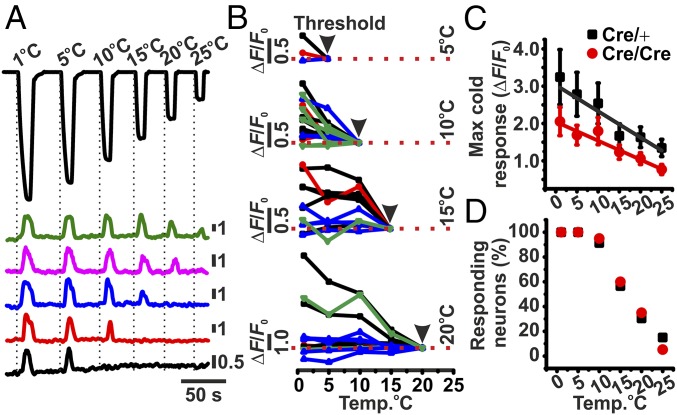
Effect of varying cooling intensities on GCaMP3 fluorescence from cold-responsive Na_V_1.8^Cre/+^ and Na_V_1.8 ^Cre/Cre^ null DRG neurons, in vivo. (*A*) Representative responses from threshold-specific cold-responsive neurons in response to a series of cooling stimuli from a holding temperature of 32 °C. (*B*) Raw traces of threshold-specific cold-responsive neurons in response to cooling stimuli of varying intensity, from 25 to 1 °C. Traces are color-coded to denote genotype and the presence (positive: +ve) or absence (negative: −ve) of tomato fluorescence (black: Na_V_1.8^Cre/+^: Tom −ve; red: Na_V_1.8^Cre/+^: Tom +ve; blue: Na_V_1.8^Cre/Cre^: Tom −ve; green: Na_V_1.8^Cre/Cre^: Tom +ve). (*C*) Average maximum fluorescent change from Na_V_1.8^Cre/+^ and Na_V_1.8^Cre/Cre^ null cold-responsive neurons in response to a series of cooling stimuli. (*D*) Percentage of cold-responsive neurons active at different temperatures. Group sizes are *n* = 4 for Na_V_1.8^Cre/+^ mice and *n* = 5 for Na_V_1.8^Cre/Cre^ null mice.

Next, we investigated how populations of threshold-specific cold-sensitive neurons responded to the intensity of the cold stimulus applied. We applied a series of transient cooling steps, ranging from 1 to 25 °C, to the plantar surface of the mouse, with each lasting 5 s, from a baseline of 32 °C. Using this protocol, we were able to identify discrete populations of cold-sensitive neurons that exhibited specific thresholds of activation, ranging from 5 to 25 °C (Fig. 3*A*). We found that neurons from each threshold group either adapted to their threshold stimulus (i.e., varying intensities of suprathreshold cold stimuli did not affect the maximal response) or exhibited a graded fluorescence response in line with the intensity of the cooling stimulus (Fig. 3*B*). However, on average, the degree of fluorescence change observed was correlated to the intensity of the cooling temperature applied (Fig. 3*C*). Interestingly, irrespective of response behavior or threshold of activation, all cold-sensitive neurons were responsive at, or below, 5 °C (Fig. 3*D*). Importantly, repeated stimulation of cold-sensitive DRG neurons to 1 °C did not change the maximal fluorescence observed (*SI Appendix*, Fig. S2 *A* and *B*). To further investigate the response profiles of individual cold-sensitive neurons, we applied a stepped cooling protocol, immediately followed by a drop cooling protocol to the plantar surface of the mouse, and measured the resulting change in GCaMP3 fluorescence in WT mice (*SI Appendix*, Fig. S3*A*). In keeping with our previous results, we were able to identify similar activation thresholds of cold-sensitive neurons, with all cold-sensitive neurons being activated at 1 °C (*SI Appendix*, Fig. S3 *B* and *C*). Furthermore, the majority of cold-sensitive neurons were small, ranging in size between 100 and 450 μm^2^, and exhibited the same threshold of activation whether stimulated by a stepped or drop cooling protocol (*SI Appendix*, Fig. S3 *B*–*E*).

### Behavioral Responses to Cold Stimuli in WT, Na_V_1.8-Null, and Na_V_1.8-Diphtheria Toxin Mice.

**Fig. 4.**
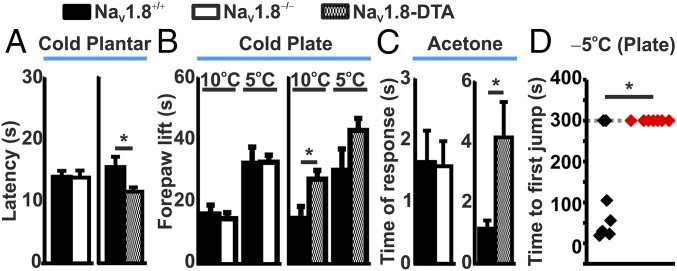
Cold-sensitivity assessment of WT, Na_V_1.8-null, and Na_V_1.8-DTA mice. (*A*) Paw-withdrawal latency of WT (*n* = 6), Na_V_1.8^−/−^ (*n* = 6), and Na_V_1.8-DTA (*n* = 6) mice in response to the cold-plantar test. (*B*) Cold-plate assessment at 10 and 5 °C of WT (*n* = 6), Na_V_1.8^−/−^ (*n* = 6), and Na_V_1.8-DTA (*n* = 6) mice. Activity was measured as the total time of forepaw lifts over the test duration. (*C*) Acetone response from WT (*n* = 6), Na_V_1.8^−/−^ (*n* = 6), and Na_V_1.8-DTA (*n* = 6) mice. (*D*) Time to first jump following placement onto a −5 °C cold plate for WT (black; *n* = 7) and Na_V_1.8^Cre/Cre^ null (red; *n* = 7) mice. A cutoff time of 300 s was used to limit tissue damage. *P < 0.05; Student’s *t* test.

To understand how the neuronal imaging results relate to behavioral responses, we performed cold-plantar, cold-plate, and acetone tests on WT and Na_V_1.8-null, as well as Na_V_1.8-diphtheria toxin (DTA) mice, where the Na_V_1.8-expressing population of sensory neurons has been ablated through the action of diphtheria toxin (11). Importantly, there was no difference in the latency of paw withdrawal in the cold-plantar test between WT and Na_V_1.8-null mice. Interestingly, however, the ablation of the Na_V_1.8 population of neurons by diphtheria toxin (DTA) caused a decrease in the latency of paw withdrawal (Fig. 4*A*). Correlating the time of paw withdrawal with the cooling properties of the glass platform (*SI Appendix*, Fig. S4), the average temperature at time of paw withdrawal was 19.61 (±1.13) °C for WT and 19.66 (±1.34) °C for Na_V_1.8-null mice. Furthermore, the deletion of Na_V_1.8 caused no change in the cold-plate response at either 10 or 5 °C, or the acetone response, compared with WT; however, the ablation of Na_V_1.8-expressing neurons led to a significantly increased response (Fig. 4 *B* and *C*). Importantly, Na_V_1.8^Cre/+^ mice displayed no difference in the cold-plate response (10 or 5 °C) compared with Na_V_1.8^Cre/Cre^ null mice (*SI Appendix*, Fig. S5*C*). Acute nocifensive responses to mechanical or heat stimulation were also performed on WT and Na_V_1.8-null mice. Although there was no difference in the latency of paw withdrawal to a noxious heat stimulus, Na_V_1.8-null mice did exhibit an increase in mechanical withdraw latency of the tail, compared with WT, consistent with previous observations (10, 12) (*SI Appendix*, Fig. S5 *A* and *B*).

### Effect of Prolonged Extreme Cold Temperatures on Behavioral and Cellular Activity.

**Fig. 5.**
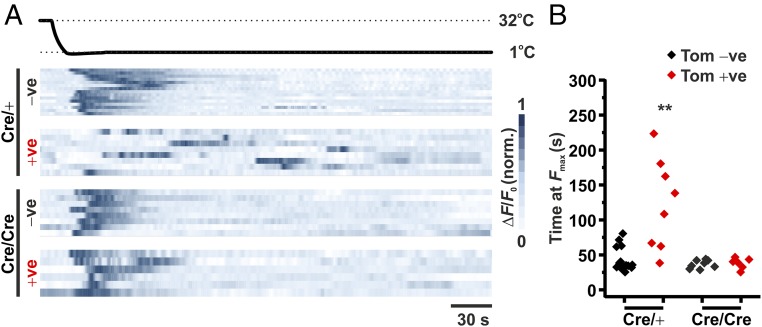
Effect of prolonged cooling on GCaMP3 fluorescence from Na_V_1.8^Cre/+^ and Na_V_1.8^Cre/Cre^ null DRG neurons, in vivo. (*A*) Heat map showing normalized changes in GCaMP fluorescence in response to a prolonged (5 min) 1 °C stimulus at the plantar surface. Individual responses from tomato negative (−ve) and positive (+ve) DRG neurons are shown, from both Na_V_1.8^Cre/+^ (*n* = 4) and Na_V_1.8^Cre/Cre^ null (*n* = 6) mice. Each row represents the response from an individual neuron. (*B*) Time at which the peak GCaMP fluorescence (*F*_max_) was recorded from the start of the prolonged cold stimulus. Values are plotted for each individual DRG neuron from each neuronal population. **P < 0.01; Kruskall–Wallis test.

Given that no difference in cold-sensing behavior was observed through imaging or behavioral analyses of WT, Na_V_1.8^Cre/+^, and Na_V_1.8^Cre/Cre^ null mice, we next investigated the effect of prolonged extreme-cold stimulation on behavioral and cellular activity. To assess the nocifensive role of Na_V_1.8 in extreme cold, mice were exposed to a −5 °C cold plate and the time taken to jump was assessed. The average time for WT mice to jump was 119 (±48.01) s; however, none of the Na_V_1.8^Cre/Cre^ null mice exhibited any jumping behavior for the duration of the assessment period (Fig. 4*D*). We next assessed the effect of extreme prolonged cooling on Na_V_1.8-expressing (+ve) and nonexpressing (−ve) DRG neurons from Na_V_1.8^Cre/+^ and Na_V_1.8^Cre/Cre^ null mice, using in vivo imaging. Extreme prolonged cold stimulation (1 °C for 5 min) caused the activation of cold-sensitive neurons within all populations of DRG neurons imaged, which typically subsided within 75 s from the initiation of the cold stimulus (Fig. 5). Within the Na_V_1.8-expressing population from Na_V_1.8^Cre/+^ mice, there was an additional population of late-responding neurons which exhibited their maximal fluorescence between 100 and 250 s from the initial cold stimulus (Fig. 5). Interestingly, late-responding neurons were not observed in Na_V_1.8-negative neurons, or in DRG from Na_V_1.8^Cre/Cre^ null mice.

### Molecular Identity of Na_V_1.8-Negative Cold-Sensitive DRG Neurons.

**Fig. 6.**
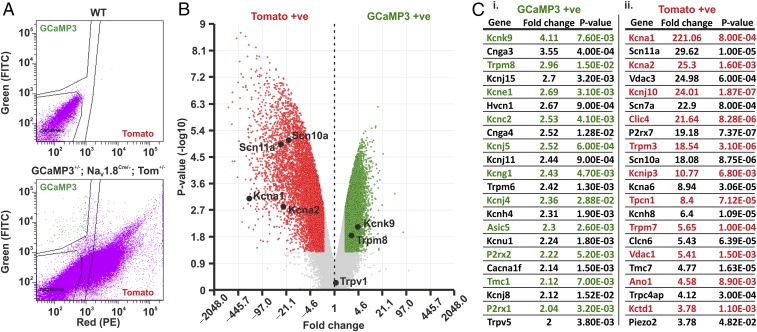
Isolation and transcriptomic analysis of putative cold-responsive GCaMP3 and tomato-positive DRG neurons. (*A*) FACS of nonfluorescent WT neurons (*Top*) and GCaMP3-Tomato expressing neurons (*Bottom*). Gating was performed to isolate GCaMP3 and tomato fluorescence, as well as to remove nonfluorescent cells. (*B*) Volcano plot showing the average fold change in gene expression versus the *P* value between GCaMP3-positive (green) and tomato-positive (red) populations. Results are filtered to genes that show a greater than twofold change in expression with a *P* value ≥0.05 (*P* < 0.05). (*C*) Summary of ion channel genes showing the greatest fold change in expression between GCaMP3 (*C*, *i*) and Tomato-positive (*C*, *ii*) populations, respectively. Cell sorting and microarray analysis was performed in triplicate (*n* = 3).

Due to our in vivo imaging and behavioral data, we wanted to investigate the identity of cold-sensitive neurons that reside outside of the Na_V_1.8-expressing population. We extracted DRG sensory neurons from mice heterozygous for Pirt-GCaMP3, Na_V_1.8 Cre, and a Cre-dependent tdTomato reporter, dissociated them, and undertook fluorescence-activated cell sorting (FACS) at 4 °C. By separating GCaMP3-only neurons from tomato-positive neurons, we were able to isolate a purified population of cold-sensitive, Na_V_1.8-negative neurons (Fig. 6 *A* and *B*). Microarray analysis of isolated RNA samples highlighted a number of gene targets that showed significant changes in expression between GCaMP3-only and tomato-positive neurons (Fig. 6*C*). Gene expression information for all genes analyzed is summarized (Dataset S1). Interestingly, the putative cold-sensing candidates *Trpm8* and *Kcnk9* showed increased expression in the GCaMP3-only population, whereas the gene encoding Na_V_1.8, *Scn10a*, as well as other ion channel genes, including *Kcna1*, *Kcna2*, *Scn11a*, and *Trpm3* showed greater expression in tomato-positive neurons. Enriched ion channel genes specific to the GCaMP3-only or tomato-only populations are summarized in Fig. 6 *C*, *i* and *ii*. Importantly, far fewer GCaMP3-only neurons were obtained when the FACS was performed at 37 °C (51 neurons), compared with 4 °C (∼342 neurons; *SI Appendix*, Fig. S6), thus supporting the use of 4 °C FACS to isolate putative cold-sensing DRG neurons.

## Discussion

Over a decade ago, the identification and characterization of Trpm8 provided a substantial mechanistic link to our understanding of how sensory neurons sense a cooling environment (13). Since then, many studies have furthered our understanding of the complex mechanisms underlying cold sensing in acute and chronic pain states, leading to the identification of numerous putative molecular candidates (14). Of these candidates, the voltage-gated sodium channel Na_V_1.8 has been identified as a major contributor to pain in cold conditions, despite showing limited overlap with Trpm8 (7). Importantly, the majority of neuronal characterization studies investigating cold sensitivity have been performed in vitro, typically involving the application of cold stimuli directly to the soma of a cultured neuron (5, 6, 13, 15). Although this approach enables a high-throughput method of screening for cold-sensing candidates, it is limited in its relevance to normal physiology, as the temperature at the level of the soma is unlikely to deviate significantly from resting core temperature. Therefore, a more instructive approach is to study cutaneous afferent sensitivity to cold by measuring the resulting neuronal activity at the level of the DRG using in vivo imaging. Using this approach, we were able to detect and record from discrete populations of cold-sensitive DRG neurons in vivo and observed that cold-sensitive neurons are organized into several threshold specific populations, reflecting the ability to sense both innocuous and noxious cold temperatures. Surprisingly, we observed that the majority of cold-sensitive neurons, activated at temperatures >1 °C, were negative for Na_V_1.8 expression, and that the deletion of Na_V_1.8 did not affect the relative number, distribution, or maximal response of cold-sensitive neurons, in vivo. Furthermore, the genetic deletion of Na_V_1.8 had no observable effect on cold-induced (>1 °C) behaviors in mice, as measured by the cold-plantar, cold-plate, or acetone tests. In contrast, nocifensive behaviors to prolonged extreme temperatures appeared to be dependent on Na_V_1.8, giving rise to the hypothesis that Na_V_1.8 is necessary for detecting cold-induced damage.

Given the essential role of Na_V_1.8 in neuronal conduction at low temperatures, it is surprising that the majority of acute cold-sensitive DRG neurons activated at temperatures ≥1 °C reside outside of the Na_V_1.8 population. The functional loss of Na_V_1.8 has been shown to prevent action potential initiation in cultured neurons at 10 °C, which is supported by a significant reduction in nocifensive behavior measured at 0 °C (5, 11). Importantly, however, despite a total loss of excitability at 10 °C from cultured neurons, the loss of Na_V_1.8 does not affect the mechanical or electrical threshold of excitability of nerve fibers. Instead, the ability of these fibers to remain active at low temperatures is dependent upon TTX-sensitive sodium channels (5). This is likely to explain our observation that the genetic deletion of Na_V_1.8 has no effect on the cold-plantar, cold-plate (10 and 5 °C), or acetone tests. In contrast, we observe that nocifensive behaviors in response to extreme cold, as measured by exposure to a −5 °C cold plate for up to 5 min, is completely absent in Na_V_1.8-null mice compared with WT mice, suggesting that while Na_V_1.8 is not required for acute cold sensing at temperatures ≥1 °C, it is required for prolonged extreme-cold stimulation. In support of this, using in vivo imaging, we show that prolonged extreme-cold stimulation causes the delayed activation of a subset of Na_V_1.8-expressing neurons, in a Na_V_1.8-dependent manner. This observation is consistent with previous data showing that the number of cold-activated nociceptors increases in line with the intensity of the stimulus applied, with up to 100% of nociceptors being activated at temperatures <0 °C (16, 17). As estimates of Na_V_1.8 expression in mouse DRG range from 68 to 85% (9, 18, 19), coupled with our observation that the majority of acute cold-sensitive neurons (activated at ≥1 °C) reside outside of the Na_V_1.8 population, it is likely that prolonged extreme-cold stimulation (≤1 °C) will principally affect Na_V_1.8-expressing neurons.

In contrast to Na_V_1.8-null mice, DTA-mediated ablation of Na_V_1.8 neurons significantly increased the cold sensitivity of affected mice at temperatures ≥5 °C. Although the explanation behind this observation is currently unclear, it was previously reported that the ablation of Trpm8 neurons distorts normal thermal responsive behavior to preferred temperatures in mice, potentially caused by reduced aversive input from the Trpm8 neuronal population (3, 4). Therefore, it is possible that the ablation of Na_V_1.8 neurons, as performed in this study, is likely to increase the relative aversive input from the surviving cold-sensing population of neurons, potentially explaining why affected mice exhibit increased aversive behavior to noxious cold stimuli.

To understand the role of specific neuronal subtypes in sensing acute noxious cold, it is important to dissociate reliably between acute innocuous and noxious cold responses. Historically, cold-responsive neurons have been categorized as being either low-threshold cold-responsive neurons (LTCR; activation range between 30 and 20 °C), or high-threshold cold-responsive neurons (HTCR; activation range from 20 to 8 °C) (20, 21). Although LTCR neurons can be regarded as innocuous cold sensors, classifying all HTCR neurons as nociceptors is more problematic, as this population is likely to contain both nociceptive and thermoreceptive afferents. One approach could be to use the paw-withdrawal threshold, a typical nocifensive response, to determine when an innocuous cooling temperature becomes noxious. Here, we show that the average temperature at which a mouse withdraws its paw is 19 °C, suggesting that all neuronal responses induced at temperature ≤19 °C can be considered noxious. Interestingly, at the level of the DRG, we found that the majority of cold-responsive neurons encode absolute threshold, rather than relative change (22), as their threshold of activation was largely unchanged when stimulated from a 32 °C drop, or by a 5 °C staircased incremental drop. Furthermore, all cold-responsive neurons were activated by all test temperatures below their activation threshold, showing either an adaptive, or a graded response to further cooling. The existence of both adaptive and graded responses supports two models for cold sensing in the periphery: a graded model where decreasing temperature causes an increase in the number and strength of neuronal responses, or a combinatorial model where the activation of distinct combinations of cold-responsive neurons encodes a specific temperature. Although the latter model is favored by some (23), without silencing either the adaptive or graded cold-responsive neurons, it is likely impossible to evaluate the physiological relevance of either population in encoding innocuous or noxious cold.

The observed threshold and coding differences of cold-sensing DRG neurons are likely to be governed by a complex repertoire of transduction mechanisms. Trpm8 is a key regulator in cold sensing, with its genetic deletion, inhibition, or cell-associated ablation causing a robust reduction in innocuous and noxious cold sensitivity (3, 4, 24). Moreover, a recent in vivo imaging study using GCaMP5 showed that Trpm8-expressing neurons encompass discrete functional classes of both innocuous and noxious cold-sensitive neurons (25). In line with these findings, we observed an enrichment for Trpm8 mRNA in our sorted cold-sensing neuronal population. In addition to Trpm8, we also observed an enrichment for the potassium channel gene Kcnk9. It has been previously observed that Kcnk9 is preferentially expressed in the Trpm8-expressing population of DRG neurons, and that its deletion causes a significant increase in cold sensitivity, consistent with potassium-channel–mediated neuronal hyperexcitability (26). In contrast, within the putative noncold sensitive, tomato-positive population, we observed, as expected, a marked enrichment in the Na_V_1.8 gene, *Scn10a*. In addition, we also observed a significant enrichment in *Kcna1* and *Kcna2* that encode the Shaker-like voltage-gated potassium channels K_V_1.1 and K_V_1.2, respectively. These channels are responsible for generating the potassium brake current (*I*_KD_), which acts to limit the excitability of sensory neurons through the generation of a voltage-dependent hyperpolarizing current (27, 28). Behavioral studies have shown that the inhibition of this current with 4-aminopyridine and alpha-dendrotoxin potently increases the cold sensitivity of mice to a 0 °C cold plate (28). Furthermore, *I*_KD_ inhibition has also been shown to mimic a cold allodynia phenotype in sham mice (27). These results suggest that, under basal conditions, *I*_KD_ reduces the excitability of cold-insensitive sensory neurons to temperatures >0 °C. Following injury, some *I*_KD_-expressing neurons become cold sensitive. This is consistent with the enrichment of another gene within the tomato-positive population, *Scn11a*, responsible for encoding the voltage-gated sodium channel Na_V_1.9, which has been shown to be a critical regulator of oxaliplatin-induced cold allodynia in the paw, while having no role in normal conditions (6).

It is clear that cold sensing relies upon a variety of distinct cellular and molecular mechanisms, and as such, a single mechanistic link for cold sensing is evidently inadequate to address the complexity of neuronal and behavioral responses to cooling. Although Trpm8 is a reliable marker for acute cold-sensing DRG neurons, other molecular candidates, such as potassium channels, are likely to contribute significantly to the fine-tuning of a neuron’s response to acute cold (28, 29). Owing to the large number of potassium channels present in DRG neurons, coupled with their essential role in the regulation of neuronal excitability, it is difficult to identify the contribution individual channels have in cold sensing. Compensation, or neuronal excitotoxicity, are likely to result from targeted knockout studies of potassium channel genes, while selective activators of different potassium channels have yet to be developed. Nevertheless, this research highlights the significant role of Na_V_1.8-negative, TRPM8-enriched sensory neurons in the detection of acute environmental cold at temperatures ≥1 °C, while prolonged extreme cold at temperatures ≤1 °C activate Na_V_1.8-postive DRG neurons.

## Methods

### Animals.

All animal procedures were approved by University College London ethical review committees and conformed to UK Home Office regulations. Experiments were performed using mice on a C57B/6 background. The following strains were used in this study: *Pirt*-GCaMP3 mice (30), Na_V_1.8^−/−^ mice (10), and Na_V_1.8-Cre mice (31). *Pirt*-GCaMP3 Na_V_1.8-Cre mice were crossed with Cre-dependent tdTomato reporter mice obtained from the Jackson Laboratory (stock no. 007905). To ablate the neuronal population of DRG neurons expressing Na_V_1.8, Na_V_1.8-Cre mice were crossed with Cre-dependent DTA mice (11).

### In Vivo GCaMP Imaging.

Na_V_1.8-Cre tdTomato GCaMP3-expressing mice (8–10 wk; mixed gender) were anesthetized using 120 mg/kg ketamine (Fort Dodge Animal Health Ltd.) and 1.2 mg/kg medetomidine (Orion Pharma). The depth of anesthesia was assessed by pedal reflexes, breathing rate, and whisker movement. Throughout the experiment the body temperature of the animal was maintained at 37 °C using a heated mat (VetTech). A dorsal laminectomy was performed at spinal level L3–L5 and the DRG was exposed for imaging as previously described (8). Artificial spinal fluid (values are in mM: 120 NaCl, 3 KCl, 1.1 CaCl_2_, 10 Glucose, 0.6 NaH_2_PO_4_, 0.8 MgSO_4_, 18 NaHCO_3_, pH 7.4 with NaOH) was constantly perfused over the exposed DRG during the procedure to maintain tissue integrity. All in vivo imaging experiments were performed using a Leica SP8 confocal microscope (Dry ×10, 0.4-N.A. objective with 2.2-mm working distance) (Leica). Bidirectional scan (800 Hz) confocal images were taken at a frame rate of 1.54 frames per s^−1^ and at a resolution of 512 × 512 pixels. The pinhole was kept at 1 a.u. Laser lines of 488 and 552 nm were used to excite GCaMP3 and tdTomato, respectively. The collection of the resulting emission for both GCaMP3 and tdTomato was system optimized to maximize yield and minimize cross-talk (Leica Dye Finder, LASX software; Leica). Laser power was <5% for both 488- and 552-nm laser lines. The stimulation was applied to the left hind paw (ipsilateral to the exposed DRG). Cold stimulation was performed by transiently immersing the hind paw with cold water (0 °C) or acetone, or by a Peltier-driven thermal stimulator (Medoc TSAII NeuroSensory Analyzer).

### Image Analysis.

All in vivo data were acquired using LASX analysis software (Leica) and analyzed using ImageJ (NIH). All images were stabilized for XY movement using the TurboReg plug-in (32), with all images being registered to the first image in a series. All fluorescent readouts were converted to Δ*F*/*F*_0_ and were analyzed as previously described (8). Where appropriate, statistical analysis was performed using repeated-measures ANOVA with Bonferroni post hoc testing, unless otherwise stated. Statistical analysis was performed in GraphPad Prism 6.0.

### Immunohistochemical Analysis.

Na_V_1.8^Cre/+^ and Na_V_1.8^Cre/Cre^ null mice were terminally anesthetized with pentobarbitone sodium (150 mg/kg; Pentoject; Animalcare). Both mouse strains also expressed the Cre-dependent tdTomato reporter gene. Whole-body fixation was then achieved through the transcardial perfusion of heparinized saline [0.1 M PBS (1× PBS) with 0.01 mL/L^−1^ heparin] followed by paraformaldehyde (4% in 1× PBS). Once fixed, whole DRG were removed from lumbar regions L3–L5 and immediately placed into a sucrose solution overnight (30% wt/vol in 1× PBS). Sections of DRG were then mounted (optimal cutting temperature embedding compound; Tissue-Tek) and sections were taken (10 μm) using a cryostat (Bright). Sections were immediately placed in washing buffer (1× PBS with 0.1% Triton 100) for 5 min, followed by 60 min in blocking buffer (1× PBS with 10% goat serum). After the blocking step, a primary antibody against NeuN (1:1,000 in 1× PBS; Sigma-Aldrich) was added to each section and incubated overnight at 4 °C. After 16 h, the incubated sections were washed (1× PBS with 0.1% Triton 100) and a secondary antibody was added (1:1,000; Alexa Fluor 488; Invitrogen) and the sections were incubated at room temperature (∼23 °C) for 1 h in the dark. The sections were then washed (1× PBS with 0.1% Triton 100) and mounted (Vectashield hard set mounting medium; Vector) for imaging. All images were taken using a Leica SP8 confocal microscope using laser lines 488 nm (NeuN:Alexa Fluor 488) and 552 nm (tdTomato).

### Behavioral Analysis.

Behavioral testing of cold sensitivity in adult mice was assessed in three different models of cold allodynia. The first model used was the cold-plantar test as described by Brenner et al. (33), and involved measuring the paw-withdrawal latency to the application of a pellet of dry ice to the glass surface (6-mm thickness). The second model was the cold-plate test. Mice were placed on an electronic cold plate (Ugo Basile) maintained at 10 or 5 °C and the total time (in seconds) that the animal spent lifting or shaking the forepaw was measured. A cutoff time of 60 s was used. For the extreme cold-plate test, mice were placed on a cold plate maintained at −5 °C for a maximum of 300 s. The time taken for each mouse to jump was assessed. Before the start of each assessment, the temperature of the cold plate was validated using an external thermometer. After jumping behavior was observed, the mouse was removed and placed onto a heated recovery plate, maintained at 37 °C, for 60 s. The third model used was the acetone sensitivity test. The mice were acclimated on the wire mesh floor in transparent plastic enclosures for at least 1 h. A drop (0.05 mL) of acetone was placed against the center of the ventral side of the hind paw. In the following 60 s after acetone application the total responding time for each mouse, as measured by paw flinching, lifting, licking, or biting, was measured (34). Mechanical nociceptive thresholds were measured using a modified version of the Randall Selitto test that applies pressure to the tail via a 3-mm^2^ blunt conical probe (35) with a 500-g cutoff. Thermal heat nociceptive thresholds were determined by measuring paw-withdrawal latency using the Hargreaves apparatus (36).

### Cell Sorting and Microarray Analysis.

DRG neurons were extracted, acutely dissociated, and resuspended in extracellular solution containing (in mM): 140 Na, 4 KCl, 1.8 CaCl_2_, 1 MgCl_2_, 10 Hepes, 5 Glucose. DNase 1 was also added to the cell suspension at 10 mg/mL Cells were immediately sorted using FACS (FACSAria III; BD Biosciences) at 4 °C, or 37 °C for control. Neuronal populations were gated based on their fluorescence. A nonfluorescent sample was used before each sorting experiment to ensure the exclusion of autofluorescent neurons. Sorted populations were immediately placed into lysis buffer and were used for RNA extraction. Cell lysis and RNA extraction was performed using the PureLink RNA Micro kit (Invitrogen). Extracted RNA was then used for microarray analysis (AffyMetrix Microarray Analysis; ThermoFisher).

## Supplementary Material

Supplementary File

Supplementary File

## References

[1] McCoy DD, Knowlton WM, McKemy DD (2011). Scraping through the ice: Uncovering the role of TRPM8 in cold transduction. Am J Physiol Regul Integr Comp Physiol.

[2] Dhaka A (2007). TRPM8 is required for cold sensation in mice. Neuron.

[3] Knowlton WM (2013). A sensory-labeled line for cold: TRPM8-expressing sensory neurons define the cellular basis for cold, cold pain, and cooling-mediated analgesia. J Neurosci.

[4] Pogorzala LA, Mishra SK, Hoon MA (2013). The cellular code for mammalian thermosensation. J Neurosci.

[5] Zimmermann K (2007). Sensory neuron sodium channel Nav1.8 is essential for pain at low temperatures. Nature.

[6] Lolignier S (2015). The Nav1.9 channel is a key determinant of cold pain sensation and cold allodynia. Cell Rep.

[7] Usoskin D (2015). Unbiased classification of sensory neuron types by large-scale single-cell RNA sequencing. Nat Neurosci.

[8] Emery EC (2016). In vivo characterization of distinct modality-specific subsets of somatosensory neurons using GCaMP. Sci Adv.

[9] Stirling LC (2005). Nociceptor-specific gene deletion using heterozygous NaV1.8-Cre recombinase mice. Pain.

[10] Akopian AN (1999). The tetrodotoxin-resistant sodium channel SNS has a specialized function in pain pathways. Nat Neurosci.

[11] Abrahamsen B (2008). The cell and molecular basis of mechanical, cold, and inflammatory pain. Science.

[12] Minett MS, Eijkelkamp N, Wood JN (2014). Significant determinants of mouse pain behaviour. PLoS One.

[13] McKemy DD, Neuhausser WM, Julius D (2002). Identification of a cold receptor reveals a general role for TRP channels in thermosensation. Nature.

[14] Lolignier S (2016). New insight in cold pain: Role of ion channels, modulation, and clinical perspectives. J Neurosci.

[15] Dhaka A, Earley TJ, Watson J, Patapoutian A (2008). Visualizing cold spots: TRPM8-expressing sensory neurons and their projections. J Neurosci.

[16] Simone DA, Kajander KC (1996). Excitation of rat cutaneous nociceptors by noxious cold. Neurosci Lett.

[17] Simone DA, Kajander KC (1997). Responses of cutaneous A-fiber nociceptors to noxious cold. J Neurophysiol.

[18] Akopian AN, Sivilotti L, Wood JN (1996). A tetrodotoxin-resistant voltage-gated sodium channel expressed by sensory neurons. Nature.

[19] Djouhri L (2003). The TTX-resistant sodium channel Nav1.8 (SNS/PN3): Expression and correlation with membrane properties in rat nociceptive primary afferent neurons. J Physiol.

[20] Nealen ML, Gold MS, Thut PD, Caterina MJ (2003). TRPM8 mRNA is expressed in a subset of cold-responsive trigeminal neurons from rat. J Neurophysiol.

[21] McKemy DD (2013). The molecular and cellular basis of cold sensation. ACS Chem Neurosci.

[22] Ran C, Hoon MA, Chen X (2016). The coding of cutaneous temperature in the spinal cord. Nat Neurosci.

[23] Wang F (2018). Sensory afferents use different coding strategies for heat and cold. Cell Rep.

[24] Almeida MC (2012). Pharmacological blockade of the cold receptor TRPM8 attenuates autonomic and behavioral cold defenses and decreases deep body temperature. J Neurosci.

[25] Yarmolinsky DA (2016). Coding and plasticity in the mammalian thermosensory system. Neuron.

[26] Morenilla-Palao C (2014). Ion channel profile of TRPM8 cold receptors reveals a role of TASK-3 potassium channels in thermosensation. Cell Rep.

[27] González A (2017). Role of the excitability brake potassium current I_KD_ in cold allodynia induced by chronic peripheral nerve injury. J Neurosci.

[28] Madrid R, de la Peña E, Donovan-Rodriguez T, Belmonte C, Viana F (2009). Variable threshold of trigeminal cold-thermosensitive neurons is determined by a balance between TRPM8 and Kv1 potassium channels. J Neurosci.

[29] Viana F, de la Peña E, Belmonte C (2002). Specificity of cold thermotransduction is determined by differential ionic channel expression. Nat Neurosci.

[30] Kim YS (2014). Central terminal sensitization of TRPV1 by descending serotonergic facilitation modulates chronic pain. Neuron.

[31] Nassar MA (2004). Nociceptor-specific gene deletion reveals a major role for Nav1.7 (PN1) in acute and inflammatory pain. Proc Natl Acad Sci USA.

[32] Thévenaz P, Ruttimann UE, Unser M (1998). A pyramid approach to subpixel registration based on intensity. IEEE Trans Image Process.

[33] Brenner DS, Golden JP, Gereau RW (2012). A novel behavioral assay for measuring cold sensation in mice. PLoS One.

[34] Flatters SJ, Bennett GJ (2004). Ethosuximide reverses paclitaxel- and vincristine-induced painful peripheral neuropathy. Pain.

[35] Randall LO, Selitto JJ (1957). A method for measurement of analgesic activity on inflamed tissue. Arch Int Pharmacodyn Ther.

[36] Hargreaves K, Dubner R, Brown F, Flores C, Joris J (1988). A new and sensitive method for measuring thermal nociception in cutaneous hyperalgesia. Pain.

