# Supplementary material for: Cold sensing by NaV1.8-positive and NaV1.8-negative sensory neurons
Source: Proc Natl Acad Sci U S A. 2019 Feb 12;116(9):3811–6. doi: 10.1073/pnas.1814545116 (PMC6397562; doi:10.1073/pnas.1814545116)
Supplement: Supplementary File [file pnas.1814545116.sapp.pdf]

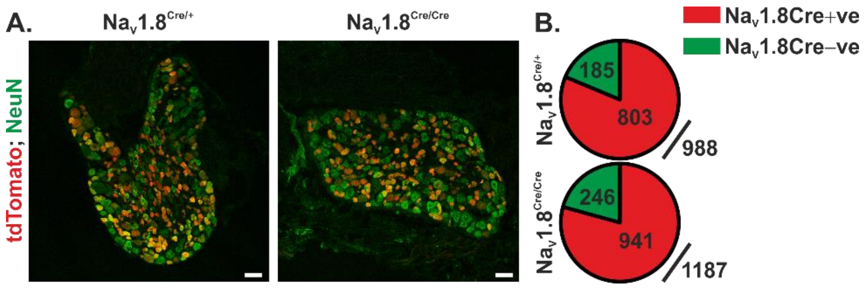

Figure S1. Immunohistochemical analysis of Cre-dependent tdTomato expression in DRG from $Na_V1.8^{Cre/+}$ and $Na_V1.8^{Cre/Cre}$ null mice. A. Example confocal images of fixed DRG slices from $Na_V1.8^{Cre/+}$ (left) and $Na_V1.8^{Cre/Cre}$ null (right) mice expressing a Cre-dependent red fluorescent reporter (tdTomato). Anti-NeuN was used to mark all DRG neurons (green). Scale bar = 100 μm. **B.** Number of tdTomato-expressing neurons as a proportion of all DRG neurons, in both $Na_V1.8^{Cre/+}$ and $Na_V1.8^{Cre/Cre}$ null mice.

www.pnas.org/cgi/doi/10.1073/pnas.1814545116

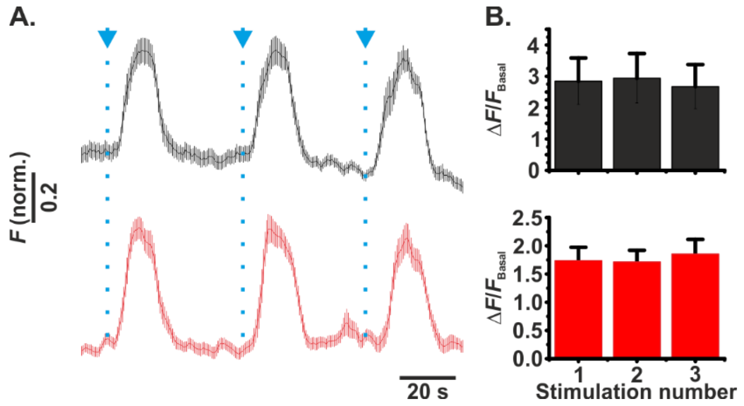

Figure S2. GCaMP responses from cold-sensitive Na$_V$1.8$^{Cre/+}$ and Na$_V$1.8 $^{Cre/Cre}$ null DRG neurons, *in vivo*, following repeated 1°C stimulation. A. Average (± S.E.M.) change in normalised GCaMP3 fluorescence in response to repeated 1°C stimulation from 32°C baseline in Na$_V$1.8$^{Cre/+}$ (black; n=23) and Na$_V$1.8$^{Cre/Cre}$ (red; n=20) DRG neurons. Blue arrowheads and dotted lines denote time of stimulus application. **B.** Average change between basal and peak fluorescence for each stimulation attempt. No significant statistical difference was observed in maximal fluorescence following repeated stimulation (repeated measures one-way ANOVA).

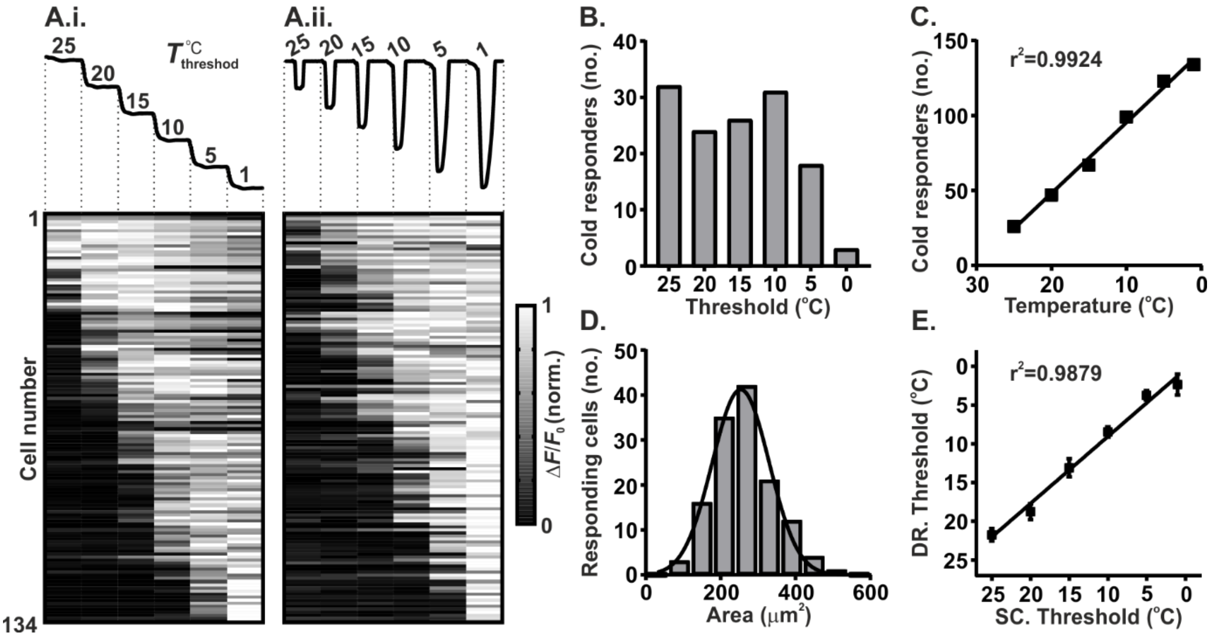

Figure S3. Temperature thresholds of DRG neuron activation in response to incremental changes in cooling. **A.** Normalised fluorescence response from cooling-sensitive DRG neurons following a staircased (A.i.) or drop temperature stimulus (A.ii.). The cooling protocols are shown at the top of the figure. Each row represents the response from the same neuron to each stimulus protocol. **B.** Summary of the threshold of DRG neuron activation observed following a staircased cooling protocol as in (A.i.). **C.** Number of DRG neurons activated by different cooling temperature drops (linear regression: $y = -4.715 * x + 142.4$). **D.** Histogram of cell area for cold-sensitive DRG neurons (Least squares Gaussian; Bin width is 60 μm^2; Mean=253.6 μm^2, Std. Dev. 76.06 μm^2). **E.** Relationship between mean thresholds of activation in response to a drop (DR) cooling stimulus versus a staircased (SC) cooling stimulus (linear regression: $y = 0.8652 * x + 0.3839$). Error bars denote S.E.M. n=134 (21 animals) for all data sets.

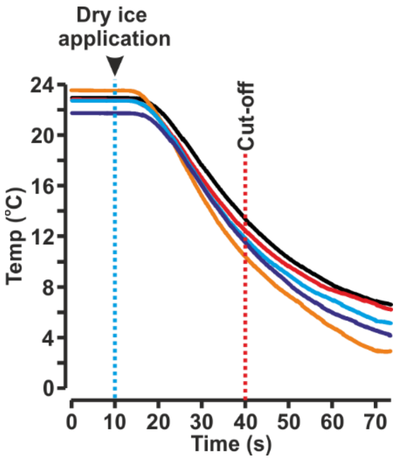

Figure S4. Cooling dynamics of cold plantar test (dry ice). Temperature of glass platform used in cold plantar test, before and during dry ice application. For behavioural testing, the maximal application time was limited to 30 seconds. Five separate trials are plotted on the graph (n=5).

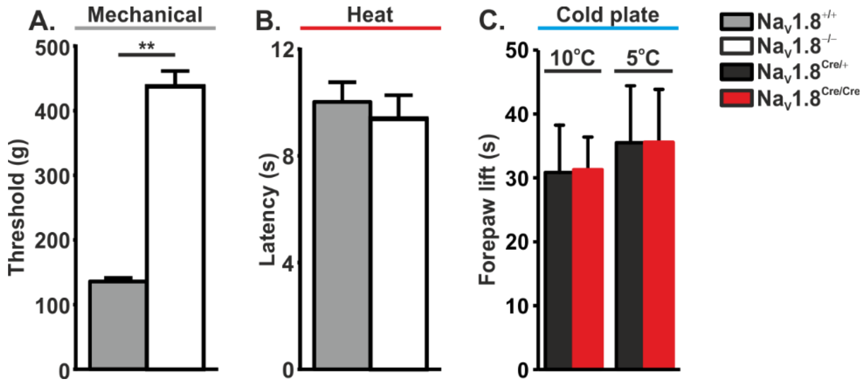

Figure S5. Acute nocifensive responses of mice with altered Na_V1.8 function. **A.** Mechanical threshold response, as measured by the Randall-Selitto test on the tail, from WT (n=6) and $Na_V1.8^{-/-}$ (n=6) mice. **B.** Heat sensitivity, as measured by the Hargreaves test, from WT (n=6) and $Na_V1.8^{-/-}$ (n=6) mice. **C.** Cold plate assessment at 10°C and 5°C of $Na_V1.8^{Cre/+}$ (n=7) and $Na_V1.8^{Cre/Cre}$ null (n=7) mice. Activity was measured as the total time of forepaw lifts over the test duration. **P<0.01; Student's T-Test.

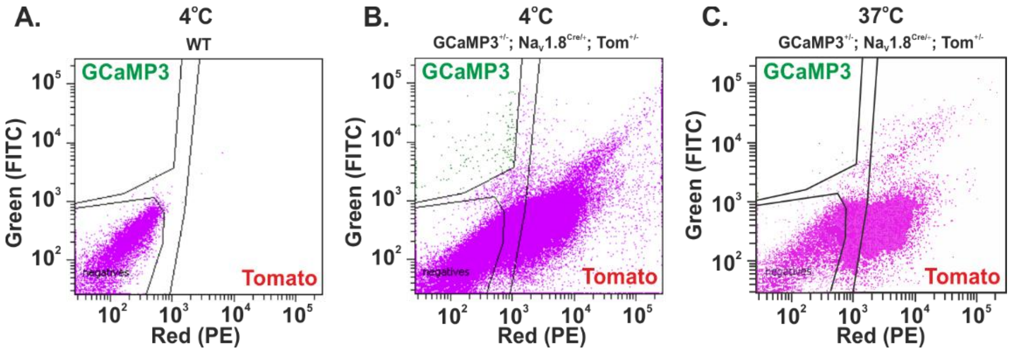

Figure S6. Fluorescence-activated cell sorting of DRG neurons at 4°C and 37°C. FACS plots of individual DRG neurons from WT (A.) and GCaMP3$^{+/-}$; Na$_V$1.8$^{Cre/+}$; Tom$^{+/-}$ (B. and C.) mice, at 4°C (A. and B.) and 37°C (C.). Gating was performed to isolate GCaMP3 and tomato fluorescence, as well as to remove non-fluorescent cells.

Supplementary dataset 1. Summary of all data obtained from the microarray analysis presented in figure 6. Averaged expression (log2) of genes from $Na_V1.8$-negative cold sensitive neurons (GCaMP3; green) and all $Na_V1.8$-positive DRG neurons (Tomato; red) following FACS, is shown (n=3). The respective fold change in gene expression between sorted neuronal populations is also shown (blue). Significance was determined at $P<0.05$ (yellow).
